# The Roles of Reward, Default, and Executive Control Networks in Set-Shifting Impairments in Schizophrenia

**DOI:** 10.1371/journal.pone.0057257

**Published:** 2013-02-27

**Authors:** James A. Waltz, Zuzana Kasanova, Thomas J. Ross, Betty J. Salmeron, Robert P. McMahon, James M. Gold, Elliot A. Stein

**Affiliations:** 1 Maryland Psychiatric Research Center, Department of Psychiatry, University of Maryland School of Medicine, Baltimore, Maryland, United States of America; 2 Neuroimaging Research Branch, National Institute on Drug Abuse-Intramural Research Program, Baltimore, Maryland, United States of America; Peking University, China

## Abstract

Patients with schizophrenia (SZ) show deficits on tasks of rapid reinforcement learning, like probabilistic reversal learning (PRL), but the neural bases for those impairments are not known. Recent evidence of relatively intact sensitivity to negative outcomes in the ventral striatum (VS) in many SZ patients suggests that PRL deficits may be largely attributable to processes downstream from feedback processing, involving both the activation of executive control task regions and deactivation of default mode network (DMN) components. We analyzed data from 29 chronic SZ patients and 21 matched normal controls (NCs) performing a PRL task in an MRI scanner. Subjects were presented with eight pairs of fractal stimuli, for 50 trials each. For each pair, subjects learned to choose the more frequently-rewarded (better) stimulus. Each time a criterion was reached, the better stimulus became the worse one, and the worse became the better. Responses to feedback events were assessed through whole-brain and regions-of-interest (ROI) analyses in DMN. We also assessed correlations between BOLD signal contrasts and clinical measures in SZs. Relative to NCs, SZ patients showed comparable deactivation of VS in response to negative feedback, but reduced deactivation of DMN components including medial prefrontal cortex (mPFC). The magnitudes of patients' punishment-evoked deactivations in VS and ventromedial PFC correlated significantly with clinical ratings for avolition/anhedonia. These findings suggest that schizophrenia is associated with a reduced ability to deactivate components of default mode networks, following the presentation of informative feedback and that motivational deficits in SZ relate closely to feedback-evoked activity in reward circuit components. These results also confirm a role for ventrolateral and dorsomedial PFC in the execution of response-set shifts.

## Background

Both the positive and negative symptoms of schizophrenia (SZ) are associated with functional disability in those who suffer from the disorder. Whereas antipsychotic medications are generally successful in attenuating the positive, or psychotic, symptoms of schizophrenia, no effective treatments exist for negative symptoms, such as motivational deficits (“avolition”) and reduced enjoyment of pleasurable activities (usually characterized as “anhedonia”). The capacity of the field to treat negative symptoms like avolition and anhedonia would likely benefit from a more detailed mechanistic understanding of these processes.

Driven by the emergence of behavioral paradigms and findings from the basic neuroscience literature, the last decade has seen a tremendous amount of research into how patients with SZ process, learn from, and decide based upon positively- and negatively-valenced outcomes. Despite the accumulation of findings pointing to deficits in reward processing and reinforcement learning in SZ, the question remains regarding the extent to which reinforcement learning abnormalities are central and/or specific to SZ, and if negative symptoms (e.g., avolition, anhedonia) have common underlying mechanisms across syndromes and illnesses (such as mood disorders).

Our primary goal was to use a well-studied behavioral paradigm (probabilistic reversal learning, or PRL) to investigate whether negative symptoms in SZ and impairments in reinforcement learning share neural substrates, suggesting common origins. The particular value of PRL paradigms is that they have been applied in numerous neuroimaging studies over the last decade, and the functional circuits underlying different components of the task are relatively well understood. Initial neuroimaging studies of PRL (e.g., [1]) pointed to a reliance of PRL on processing in ventral prefrontal cortex (PFC) and ventral striatum (VS). Given the dependence of PRL performance on the integration and correction of errors, these findings accord with the identified roles for these structures in the sensitivity to negative feedback (in ventrolateral prefrontal cortex, or vlPFC, in particular) and in the computation of mismatches between anticipated and obtained outcomes, called reward prediction errors, or RPEs (in the VS, especially) [2]–[5]. More recent work, however, indicates that successful PRL depends upon multiple neural circuits involved in reward processing and decision making, with component structures including dorsolateral prefrontal cortex (dlPFC), dorsomedial prefrontal cortex (dmPFC), and amygdala [6], all of which are highly interconnected with vlPFC and VS [7]–[9].

We previously conducted a behavioral study with a PRL paradigm, the results of which pointed to particular problem of reversing learned associations in SZ [10]. This behavioral finding led us to conjecture that PRL deficits in SZ emerge, at least in part, from an abnormal sensitivity to negative feedback, as well as dysfunction in signaling mismatches between anticipated and obtained outcomes (RPEs), brought on by dysfunction in vlPFC and VS.

However, PRL deficits need *not* stem directly from a reduced sensitivity to either positive or negative outcomes. For example, SZ patients would also achieve fewer reversals in a probabilistic environment if they were *overly-sensitive* to punishments (some of which would be invalid), and thus shifted too readily (i.e., showed difficulty in maintaining set following rewards). Alternatively, even if SZ patients showed normal sensitivity to positive or negative feedback, they might have difficulty in *using* that feedback to drive subsequent behavior, either because they had a reduced ability to update representations of the value of stimuli and actions following feedback [11] or because they had a reduced ability to appropriately modulate attention (to subsequent stimuli, actions, and feedback) following surprising punishments. A reduced ability to update representations of the value of stimuli and actions following feedback would likely involve dysfunction primarily in ventral and medial PFC regions, such as pregenual and dorsal anterior cingulate cortex [12], [13].

Based on our previous behavioral findings [14], [15], we predicted that SZ patients would show impaired PRL performance, relative to controls, brought on by both non-normative lose-shift behavior and non-normative win-stay behavior. That is to say: we expected SZs to be both less likely than controls to shift to the response-alternative after a punishment, and less likely than controls to stay with the current response after a reward. Based on our previous neuroimaging finding of abnormal prefrontal responses to monetary losses in SZ patients [16], but intact striatal responses, we predicted that impaired PRL performance in SZs would be accompanied by reduced differentiation between rewards and punishments in cortex (vmPFC), though not necessarily in striatum. Finally, based on our previous finding [16], we hypothesized that sensitivity to feedback valence in striatum would be predictive of negative symptom severity, especially avolition and anhedonia, though not necessarily of a diagnosis of schizophrenia itself.

A second major goal of the work was to examine the contributions of broader networks (those in addition to brain reward circuits) to deficits in reinforcement learning and motivation in SZ. Critically, recent research has revealed that the ability to carry out goal-directed behavior involves both the activation of so-called task-positive regions and the suppression of activity in so-called task-negative regions [17]–[19]. A failure to adaptively modulate attention based on the unexpectedness of feedback, then, would likely be associated not only with a reduced ability to engage task network components (also known as executive control networks, ECN), but also with a reduced ability to suppress activity in task-negative regions, such as default mode network (DMN) structures [17], [19], [20]. The DMN is comprised of a set of brain regions that typically deactivate during performance of cognitive tasks [21] and is often identified using task-free functional connectivity MRI [22]. Thus, one would expect reduced DMN deactivation during task performance to be a neural correlate of a failure to adaptively modulate attention based on the salience of feedback. In the current study, we examined: 1) the differential deactivation of DMN nodes (relative to baseline) across conditions, as signals of the ability of stimuli to modulate attention; and 2) group differences in contrasts in DMN activations in response to stimuli and events, as indicators of the relative abilities of patients and controls to modulate attention according to the salience of stimuli and events.

In fact, evidence indicates that DMN deactivation figures critically in attentional-set-shifting [23]–[25]. Apart from reduced general attention-related DMN deactivation, reduced *task-specific* deactivations have been observed in SZ patients in the context of cognitive task performance and attributed to DMN dysfunction [26]–[28]. Additional research [29] has found reduced segregation among default mode and executive control networks at rest, a fact which may explain a reduced ability to adaptively suppress DMN activity during task performance in SZ. We specifically wanted to investigate: 1) whether SZ patients showed reduced task-related deactivations in the context of RL (that is, whether they showed reduced deactivations in response to salient *feedback*); and 2) whether reduced task-related deactivations were observed in SZ in *independently-identified* DMN structures. To this end, we quantified task-related DMN suppression, after first identifying a set of DMN regions of interest (ROIs), through a separate analysis of resting state connectivity in the same subjects, in the same session.

Based on evidence of well-established deficits in SZ patients in ECN network function in the context of cognitive control tasks [30], we predicted that SZ patients would exhibit abnormal responses to negative feedback preceding choices to switch in dmPFC, dlPFC, and vlPFC. We further hypothesized that, in addition to reduced task-related ECN activations, SZ patients would also exhibit attenuated responses (reduced deactivations) to unexpected outcomes in DMN components, such as vmPFC and posterior cingulate cortex (PCC).

## Methods

### Recruiting and Screening of Participants

Thirty-five patients and 23 healthy control subjects, matched on demographic characteristics and smoking status, underwent MRI scanning. All participants were right-handed, as determined by the Edinburgh Handedness Inventory [31], and provided written informed consent to protocols approved by the Institutional Review Boards of the National Institute on Drug Abuse's Intramural Research Program (Protocol 05-DA-N401) and the University of Maryland School of Medicine (Protocol HP-00042701). To ensure understanding of the study, all participants with a diagnosis of schizophrenia were administered the Evaluation to Sign Consent (ESC) [32], a short questionnaire about study demands and risks, as well as subject rights. No patient was enrolled in the study without first demonstrating adequate performance (at least 10 points out of a possible 12) on the ESC. All patients were on stable antipsychotic medication regimens (no changes for four weeks), all with second-generation antipsychotics (SGAs). The diagnosis of schizophrenia or schizoaffective disorder in patients was confirmed using the SCID-I [33], as was the absence of Axis I diagnoses in control participants. Control participants diagnosed with Axis II personality disorders, based on screening with the SIDP-R [34], were also excluded. All participants underwent medical screening, involving a medical history and physical exam. Exclusionary criteria included: pregnancy, current illegal drug use (both verified by urine screens), admission of past substance dependence, and any neurological or medical illness that might confound data interpretation. Participants were instructed to abstain from alcohol for 24 hours prior to study visits (verified by a breathalyzer); smokers were allowed to smoke prior to MRI scanning, so as to avoid potential effects of nicotine withdrawal.

### General Procedures

Outside of the MRI scanner, cognitive function was assessed using three standard measures: the Wechsler Abbreviated Scale of Intelligence (WASI) [35], the Wechsler Test of Adult Reading (WTAR) [36], and the Repeatable Battery for the Assessment of Neuropsychological Status (RBANS) [37], [38]. In order to assess the extent to which all study participants experience pleasure both physically and in social contexts, all subjects completed the Scales for Physical and Social Anhedonia [39]. Standard symptom ratings were obtained for all patients using the Scale for the Assessment of Negative Symptoms (SANS) [40], the Brief Psychiatric Rating Scale (BPRS) [41], and the Calgary Depression Scale (CDS) [42].

### Probabilistic Reversal Learning (PRL) task

**Figure 1:**
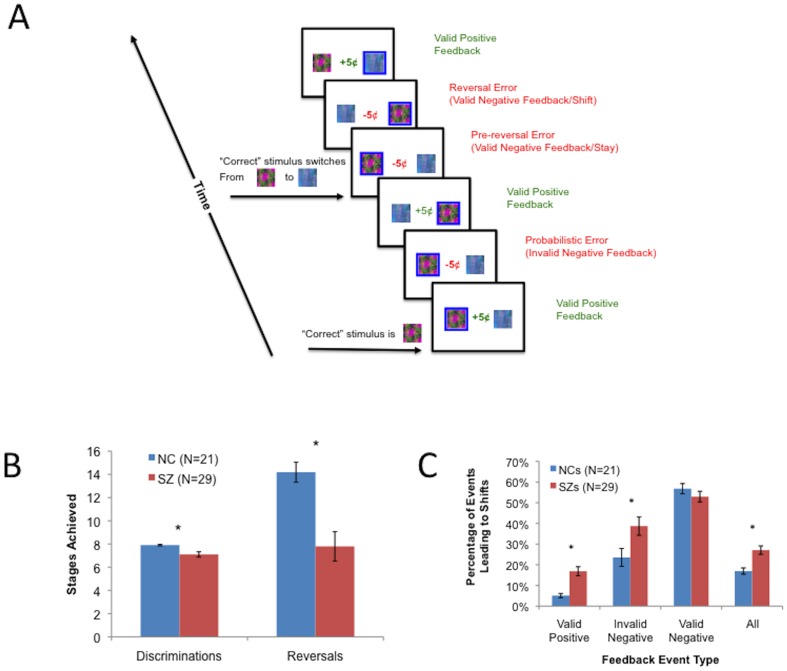
Probabilistic Reversal Learning task and results. (**A**) Probabilistic Reversal Learning task, showing types of feedback events. (**B**) RL task Discrimination and Reversal stages achieved in SZ patients and controls. (**C**) Proportions of different types of RL task events leading to performance shifts.

In order to investigate processes involved in feedback-driven learning and choice, we administered a PRL task based on Cools et al. [1], in conjunction with functional magnetic resonance imaging (fMRI; see Figure 1A). Prior to MRI scanning, subjects were instructed on how to perform the task on a desktop computer, before undergoing a training session in a mock scanner (see Supporting Information for details on instructions and training). In the MRI scanner, subjects were presented with eight blocks of 50 trials, each with two unique fractal patterns. Trials were separated in time by a variable ITI (range: 1–3 s; mean: 2 s). The left/right ordering of the stimuli was randomized, and the stimuli appeared for 2 seconds. Once a choice was made by a left or right button-press, a blue frame surrounded the chosen fractal for the duration of the exposure (2 s-RT), with feedback presented at fixation (either +5¢ in green, or -5¢ in red). In each pair, the choice of one pattern resulted in a gain 80% of the time and a loss 20% of the time, while the other resulted in a gain 20% of the time and a loss 80% of the time (termed an 80%/20% discrimination). When subjects achieved a criterion of nine choices of the better stimulus in a run of ten trials, the infrequently rewarded stimulus became the better choice, and the frequently rewarded stimulus became the worse choice.

Subjects achieved as many reversals as they could within a block of 50 trials, after which they were presented with the next pair and required to learn a new discrimination, with the same reinforcement probabilities. The purpose of presenting subjects with eight separate probabilistic discriminations to learn was to enable us to contrast performance in initial discrimination learning with performance during attempts to reverse the initial discrimination, once learned. It also limited the number of incorrect trials experienced by subjects before they were given a chance to learn a new discrimination. Subjects who achieved fewer than 5 (of the 8 possible) discriminations or performed fewer than 50 reversal trials (trials after achieving criterion on the initial discrimination in a block) *during the MRI scanning session* were excluded from further analyses. These criteria applied to six patients and two controls; thus 29 patients and 21 controls were included in group analyses of subject behavioral and MRI data (see Table 1 for subject details).

**Table 1:** Subject characterizing information.

		Patients	(N = 29)	Controls	(N = 21)	p of Group Diff.
*Demographics*						
	Age	39.6	(10.0)	39.6	(10.5)	ns
	Gender	5 F,	24 M	6 F,	15 M	ns
	Race	18 W,	11 NW	14 W,	7 NW	ns
	Smokers	11 Y,	18 N	6 Y,	15 N	ns
	Subject Education (years)	13.4	(1.7)	15.1	(2.1)	0.003
	Parental Education (years)	14.2	(3.4)	14.2	(3.3)	ns
*Neuropsychological Testing/Questionnaires*						
	IQ (from WASI 4-subtest)	102.9	(13.6)	116.6	(11.8)	0.001
	WTAR Scaled Score	101.3	(17.1)	109.7	(11.7)	0.044
	RBANS Total	86.3	(15.5)	103.8	(9.2)	<0.001
	Chapman–Phys. Anhed.	14.6	(9.1)	10.8	(9.4)	ns
	Chapman–Soc. Anhed.	11.9	(7.4)	9.8	(6.3)	ns
*RL Measures*						
	Discrimination Error%	24.1	(11.0)	13.7	(5.7)	<0.001
	Reversal Error%	35.1	(11.3)	24.9	(5.7)	<0.001
	Overall Shift%	27.1	(10.9)	17.0	(6.9)	<0.001
	Valid Positive Shift%	16.9	(11.8)	5.1	(4.6)	<0.001
	Invalid Negative Shift%	38.7	(23.9)	23.6	(19.9)	0.022
	Valid Negative Shift%	52.9	(13.9)	56.8	(11.3)	0.301
*Clinical Characteristics*						
	BPRS Total Score	37.7	(6.4)			
	Sum of Global Scores	5.8	(3.5)			
	from four SANS subscales					
	Antipsychotic Medications					
	-Clozapine	N =	11			
	-Risperidone	N =	8			
	-Olanzapine	N =	4			
	-Quetiapine	N =	3			
	-Ziprasidone	N =	1			
	-Risp+Olanz	N =	2			

Abbreviations: Diff., Difference; ns, non-significant; F, Female; M, Male; W, White; NW, Non-white; WASI, Wechsler Abbreviated Scale of Intelligence; WTAR, Wechsler Test of Adult Reading; RBANS, Repeatable Battery for the Assessment of Neuropsychological Status; Phys., Physical; Anhed., Anhedonia; Soc., Social; BPRS, Brief Psychiatric Rating Scale; SANS, Scale for the Assessment of Negative Symptoms; Risp, risperidone; Olanz, Olanzapine. Numbers in parentheses are standard deviations.

### Analyses of behavioral data

In addition to its valence, we considered two additional aspects of feedback: 1) whether the feedback was valid or not (depending on whether the better stimulus was rewarded or punished); and 2) whether the feedback prompted the subject to "stay" with the current stimulus on the next trial, or "shift" to the alternative. Based on these two properties of feedback, we considered three classes of negative feedback events, or "errors", as defined by [1] (see Figure 1A): 1) instances of "invalid" negative feedback (when a subject was punished for choosing the better stimulus; termed "probabilistic errors" by [1]); 2) instances of "valid" negative feedback leading to shifts (when a subject was punished for choosing the worse stimulus, and subsequently shifted to the alternate stimulus; termed "final reversal errors" by [1]); and 3) instances of "valid" negative feedback leading to stays (when a subject was punished for choosing the worse stimulus, but then chose the same stimulus again; termed "preceding reversal errors" by [1]). A response was considered to be “correct” if it involved the choice of the more frequently rewarded stimulus (not necessarily if it involved positive feedback). Therefore, choices of the more frequently rewarded stimulus resulting in negative feedback (probabilistic errors) were actually considered to be correct.

Behavioral measures submitted to second-level analyses included initial discriminations achieved, total reversals achieved, the percentage of incorrect responses made in learning initial discriminations, and the percentage of incorrect responses made *following* learning of initial discriminations. We used Mann-Whitney U-tests to examine group differences in initial discriminations achieved and reversals achieved and t-tests to examine group differences in percentages of incorrect responses made in the discrimination and reversal phases. Independent-samples t-tests examined effects of diagnostic group on subjects' rates of switching to the alternate stimulus evoked by the following feedback events: 1) any kind of feedback; 2) valid negative feedback (called Valid Lose-Shifts, or Appropriate Shifts); 3) invalid negative feedback (called Invalid Lose-Shifts); and 4) valid positive feedback (called Valid Win-shifts).

### MRI data acquisition

In conjunction with the performance of the PRL task, whole-brain functional EPI images were acquired using a 3-T Siemens Allegra scanner (Erlangen, Germany) for measurement of T2*-weighted BOLD effects (64×64 matrix; FOV = 22×22 cm; TR = 2 s; TE = 27 ms; FA = 80°). In order to reduce susceptibility artifacts [43], [44], we used 4-mm oblique axial slices, 30° axial to coronal. We acquired 213 volumes in each of four runs, and each run consisted of two blocks of 50 trials presented serially (each block with a different pair of fractal patterns). The four EPI scans thus lasted about 28.5 minutes and were accompanied by 400 task trials. Head motion was minimized using foam padding or foam molding, and in each scanning session, a whole-brain oblique axial T1-weighted structural image (MPRAGE) was acquired for anatomical reference (1-mm^3^ isotropic voxels; TR = 2.5 s; TE = 4.38 ms; FA = 8°).

Resting state MRI data were collected from 49 of the 50 subjects with usable probabilistic reversal learning (PRL) task data during the same session as the functional data. During the resting scans, subjects were given a simple instruction to rest and keep their eyes open. A static neutral image (the projector's logo) was presented on the screen during the resting scan. Resting-state fMRI were acquired with the same EPI sequence using for the event-related data (150 volumes), immediately after performance of the PRL task.

### Preprocessing of resting state MRI data

Data were analyzed in AFNI [45] and the SPM toolbox [46] for Matlab (The MathWorks, Inc.; Natick, MA). Volumes were slice-timing aligned and motion corrected to the base volume that minimally deviated from other volumes using an AFNI built-in algorithm. After linear de-trending of the time course of each voxel, volumes were spatially normalized and resampled to Talairach space at 3×3×3 mm^3^, spatially smoothed (FWHM, 6 mm), and temporally low-pass filtered (f_cut-off_ = 0.1 Hz).

### Identification of Default Mode Network ROIs

The DMN was identified from functional connectivity analyses of resting state data, using a PCC seed comprised of a 10-mm radius sphere centered on left PCC (coordinates: −5, −49, 40, as specified by [47], one of the canonical papers on the identification of distinction neural processing networks using functional MRI). Correlation analyses were performed by calculating the cross-correlation coefficient (CC) between the PCC time course extracted by averaging time courses of all of the voxels in the PCC ROI and the time courses of each voxel of the brain, including the six rigid head-motion parameter time courses, the average time course in white matter, and average time course in cerebrospinal fluid as nuisance covariates [47], [48]. A white matter mask was generated by segmenting the high-resolution anatomical images in SPM5 and down-gridding the obtained white matter masks to the same resolution as the functional data [49]. These nuisance covariates regress out fluctuations likely to be irrelevant to neuronal activity [47]. For each participant, a PCC-seed-based whole-brain CC resting-state functional connectivity (rsFC) map was generated and then transformed to a z-map based on the Fisher z-transformation, a step that identified the functional connectivity of the PCC. Two-tailed one-sample t-tests were performed on the z-score maps to obtain group rsFC maps of the PCC at p<0.05, corrected (voxel-wise threshold of p<0.001) [50], for: 1) controls only; 2) SZ patients only; and 3) the entire sample. Coordinates of the centers of mass of resulting ROIs are shown in Table 2. Finally, we compared rsFC values in these regions across subjects groups, using independent samples t-tests (again using a threshold of p<0.05, corrected).

**Table 2:** Results of Resting State Functional Connectivity (rsFC) analyses.

		All Subjects						NCs Only						SZs Only					
		x	,	y	,	z	Vol	x	,	y	,	z	Vol	x	,	y	,	z	Vol
Location	L/R	(R+	,	A+	,	S+)	(µl)	(R+	,	A+	,	S+)	(µl)	(R+	,	A+	,	S+)	(µl)
mPFC	L	−21	,	63	,	18	648	−5	,	45	,	12	2727						
mPFC	R	10	,	58	,	8	4590	3	,	60	,	−2	918						
Sup. Frontal G.	L							−27	,	62	,	12	729						
Sup. Frontal G.	L	−29	,	19	,	47	4212	−38	,	20	,	42	1377	−28	,	19	,	48	1296
Sup. Frontal G.	R							21	,	62	,	9	1269	26	,	57	,	11	567
Sup. Frontal G.	R	23	,	26	,	49	8775	26	,	27	,	46	4725	23	,	27	,	50	5805
Supramarginal G.	L	−46	,	−64	,	32	16578	−47	,	−64	,	35	9585						
Supramarginal G.	R	48	,	−60	,	32	20061	50	,	−59	,	35	12042	47	,	−62	,	31	18090
Fusiform G.	R							33	,	−71	,	−12	675						
PCC	L/R	−1	,	−52	,	34	69012	−1	,	−52	,	35	52704	−6	,	−55	,	33	79704

Abbreviations: NCs, normal controls; SZs, patients with schizophrenia; Vol, volume; BA, Brodmann Area; L, left; R, right; A, anterior; S, superior; mPFC, medial prefrontal cortex; Sup., superior; G., gyrus; PCC, posterior cingulate cortex.

### Preprocessing of Event-related Data

All preprocessing and first-level analyses of MRI data were performed using the AFNI software package [45]. Preprocessing steps included volume-registration for motion correction, slice-timing correction, temporal normalization, and blurring to a full-width, half-maximum of 8 mm. Regressors in general linear models (GLMs) of single-subject time series included three types of negative feedback events (valid lose-stays, valid lose-shifts, and invalid instances of negative feedback) and three types of positive feedback events (valid win-stays, valid win-shifts, and invalid instances of positive feedback). Regressors were delta-functions time-locked to the onset of the aforementioned events, convolved with a model hemodynamic response function (HRF) and its temporal derivative. Further regressors included non-responses, as well as head-motion curves to help account for residual motion effects.

### Whole-brain Analyses of Event-related MRI Data

First, based on the work of Cools et al. [1], we contrasted beta coefficients for valid instances of negative feedback leading to shifts with those for valid instances of positive feedback leading to stays. We then identified neural activity changes specific to valence by contrasting beta coefficients for valid instances of negative feedback with those for valid instances of positive feedback, both leading to stays (thereby controlling for the ensuing decision). Finally, we identified neural activity changes specific to final reversal errors (instances of valid feedback actually evoking shifts) by contrasting beta coefficients for valid instances of negative feedback evoking shifts with those evoking stays (thereby controlling for the type of feedback). This contrast was also inspired by Cools et al. [1], who isolated neural activity specific to feedback responses that prompted reversals to the alternate stimulus. For each of these contrasts, we performed two-way linear mixed effect (LME) analyses on single-subject average parameter estimates, with factors of GROUP (patients vs. controls) and EVENT-TYPE. Monte Carlo simulations determined that a voxel-wise threshold of p<0.001, together with a minimum cluster size of 21 voxels (567 µl), was required to achieve a significance level of p<0.05, correcting for multiple comparisons over the whole brain.

### Contrasts in Regions-of-Interest

The investigation of feedback-evoked responses in DMN areas was motivated by previous findings of reduced DMN suppression during task performance [26], [27]. Because the DMN ROIs were established independently of the whole-brain event-related analyses, we performed two-way ANOVAs to examine effects of GROUP and VALENCE on feedback-evoked responses in these regions. We also performed two-way ANOVAs to examine effects of GROUP and BEHAVIOR (lose-shift vs. lose-stay) on feedback-evoked responses in DMN ROIs. Finally, based on Cools et al. [1], we performed two-way ANOVAs to examine effects of both GROUP and VALENCE (lose-stay vs. win-stay) and GROUP and BEHAVIOR (lose-shift vs. lose-stay) on feedback-evoked responses in left and right VS, using coordinates (±10, 8, -4; radius 8) from the original study of PRL by this group [1]. Coordinates of additional components of reward (vmPFC), salience (vlPFC), and executive control (dlPFC, dmPFC) were drawn from the results of whole-brain contrasts reported below.

### Correlational Analyses using ROIs

In order to examine more closely how symptom severity modulated feedback-related responses, we performed correlation analyses involving contrasts between neural responses to different types of feedback events in the same set of brain regions described in the preceding paragraph. For all ROIs, Pearson correlation analyses assessed relationships among subscale scores for avolition/anhedonia in SZ patients, and BOLD signal contrasts in the above-mentioned ROIs. We computed the avolition/anhedonia factor score by summing global scores for avolition/role-functioning and anhedonia/asociality from the SANS. To characterize the influence of antipsychotic drugs (APDs) on network activity, Pearson correlation analyses assessed relationships between BOLD signal contrasts in ROIs and APD doses for SZ patients (converted to haloperidol equivalents, using the method of [51]) and BOLD signal contrasts in ROIs.

## Results

### Behavioral Data

Schizophrenia patients achieved both fewer initial discriminations (p = 0.006) and reversals than controls (p<0.001; see Figure 1B). T-tests (Table 1) revealed that patients with SZ also made more errors than controls in both discrimination and reversal stages, and that SZ patients showed greater overall rates of shifting, as well as greater tendencies to switch in response to both positive feedback and probabilistic negative feedback (Figure 1C).

### Resting-state Functional Connectivity (rsFC) Analyses

When we examined rsFC in control subjects, we identified ten brain areas showing significantly correlated activity with the PCC seed (Table 2). These areas included clusters in medial PFC, left and right posterior parietal cortex (PPC), left and right superior frontal gyrus, and an extended region of PCC (Figure S1A–B). Patients also showed significant rsFC between the seed region and lateral PPC and superior frontal gyrus (along with a large region of PCC; Table 2). They did not show significant rsFC between the seed region and mPFC (Figure S1C–D). A 2-sample t-test revealed no group differences between the rsFC maps for patients and controls (Table 2). The rsFC map that was common to both patients and controls (generated from a 1-sample t-test using individual maps from the 49 subjects completing the resting MRI scan) included clusters in medial PFC, left and right PPC, left and right superior frontal gyrus, and a large region of PCC/precuneus (Table 2; Figure S1E–F). These seven regions were submitted to subsequent ROI analyses of event-related BOLD responses from the PRL task.

### Whole-brain Analyses: Lose-shifts vs. Win-stays

**Figure 2:**
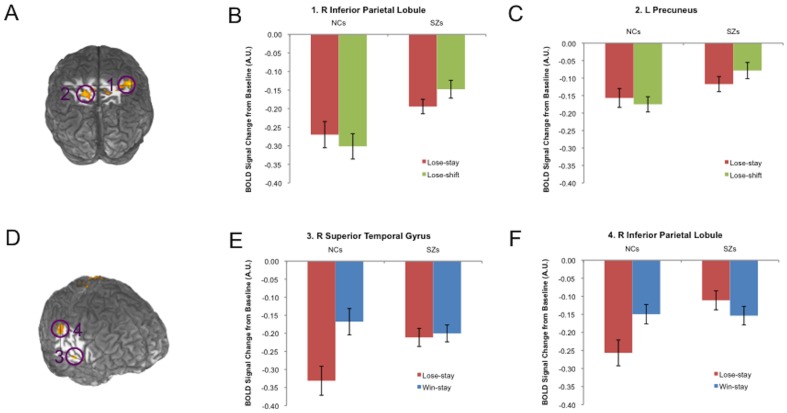
Brain regions showing group differences in condition contrasts (GROUP and CONDITION interactions). (**A**) Interacting effects of GROUP and CONDITION (lose-shift vs. win-stay) on responses were observed in R inferior parietal lobule and left precuneus (brain cuts at y = −33 and Z = 56). In both (**B**) R inferior parietal lobule and (**C**) left precuneus, controls showed much stronger deactivations for lose-shifts (relative to win-stays) than patients did. (**D**) Interacting effects of GROUP and CONDITION (lose-stay vs. win-stay) on responses were observed in R lateral temporal cortex and R inferior parietal lobule (brain cuts at X = 58, Y = −29, Z = 0). In both (**E**) R lateral temporal cortex and (**F**) R inferior parietal lobule, controls showed much stronger deactivations for negative (relative to positive) feedback than patients did, when both forms of feedback led to stays.

When we contrasted valid instances of negative feedback leading to shifts with those for valid instances of positive feedback leading to stays, the two-factor LME model revealed two brain regions showing GROUP×CONDITION (lose-shift vs. win-stay) interactions at a corrected threshold of p<0.05: right inferior parietal lobule (IPL/BA 40) and left precuneus (BA 7; Table 3; Figure 2A). Each of these regions showed greater BOLD signal contrasts between lose-shifts and win-stays in controls than patients, driven by greater deactivations for lose-shifts in the control group (Figure 2B–C).

**Table 3:** Brain regions showing group differences in condition contrasts.

Brain regions showing group differences in the [Lose-shift-Win-stay] contrast										
				x	,	y	,	z		
Brain Region (Cluster #)		BA	L/R	(R+	,	A+	,	S+)	Voxels	Vol (µl)
1.	Inferior Parietal Lobule	40	R	42	,	−33	,	54	93	2511
2.	Precuneus	7	L	−18	,	−51	,	57	49	1323

Brain regions showing group differences in the [Lose-stay-Win-stay] contrast										
				x	,	y	,	z		
Brain Region (Cluster #)		BA	L/R	(R+	,	A+	,	S+)	Voxels	Vol (µl)
3.	Superior Temporal G.	22	R	54	,	1	,	0	24	648
4.	Inferior Parietal Lobule	40	R	62	,	−29	,	32	24	648

Brain region numbers correspond to those illustrated in Figure? 3.

Abbreviations: BA, Brodmann Area; L, left; R, right; A, anterior; S, superior; Vol (µl), volume in microliters; G., gyrus.

We found that main effects of feedback VALENCE were of two types: those showing greater activation for lose-shifts than win-stays, and those showing the opposite pattern. As shown in Table 4, areas showing greater activation in response to lose-shifts than to win-stays included vlPFC and PPC, brain regions often linked to action selection and inhibition [52], [53]. In contrast, areas showing greater activity in response to win-stays vs. lose-shifts included structures along the midline (middle and posterior cingulate), as well as superior frontal gyrus and lateral temporal cortex.

**Table 4:** Brain regions showing significant [Lose-shift-Win-stay] contrasts.

Lose-shift>Win-stay										
				x	,	y	,	z		
Brain Region (Cluster #)		BA	L/R	(R+	,	A+	,	S+)	Voxels	Vol (µl)
	*Frontal Cortex/Midbrain*									
1.	Insula/vlPFC	13	L	−39	,	12	,	6	161	4347
2.	Insula/vlPFC^‡^	13	R	33	,	18	,	9	2448	66096
3.	Midbrain		R	6	,	−24	,	−6	33	891
	*Temporal/Parietal Cortex*									
4.	Inferior Parietal Lobule	7	R	35	,	−58	,	46	22	594
5.	Parahippocampal G.		R	27	,	−54	,	−6	212	5724
	*Occipital Cortex*									
6.	Middle Occipital G.		R	33	,	−84	,	15	1165	31455
	*Cerebellum*									
7.	Culmen		L	−33	,	−45	,	−18	58	1566
8.	Culmen		L	−36	,	−51	,	−30	23	621

Lose-shift < Win-stay										
				x	,	y	,	z		
Brain Region (Cluster #)		BA	L/R	(R+	,	A+	,	S+)	Voxels	Vol (µl)
	*Frontal Cortex*									
9.	Superior Frontal G.	10	L	−15	,	60	,	21	1096	29592
10.	Superior Frontal G.	8	L	−18	,	42	,	42	29	783
11.	Middle Frontal G.	47	L	−42	,	39	,	−6	26	702
12.	Superior Frontal G.	8	L	−30	,	21	,	51	57	1539
13.	Cingulate G.		R	18	,	−6	,	27	59	1593
	*Temporal/Parietal Cortex*									
14.	Superior Temporal G.	22	R	57	,	−3	,	3	36	972
15.	Inferior Temporal G.	21	L	−60	,	−12	,	−15	77	2079
16.	PCC		R	−9	,	−57	,	15	282	7614
	*Occipital Cortex*									
17.	Superior Occipital G.		L	−45	,	−78	,	27	163	4401

Abbreviations: BA, Brodmann Area; L, left; R, right; A, anterior; S, superior; Vol (µl), volume in microliters; vlPFC, ventrolateral prefrontal cortex; G., gyrus; PCC, posterior cingulate cortex.

### Whole-brain Analyses: Lose-stays vs. Win-stays

When we contrasted valid instances of negative feedback leading to *stays* with those for valid instances of positive feedback leading to stays, the two-factor LME model revealed two brain regions showing GROUP×CONDITION (lose-stay vs. win-stay) interactions at a corrected threshold of p<0.05: right lateral temporal cortex and right inferior parietal lobule (IPL/BA 40; Table 3; Figure 2D). Each of these regions showed greater BOLD signal contrasts between positive and negative outcomes in controls than patients, driven by greater deactivations for surprising punishments in the control group (Figure 2E–F).

**Figure 3:**
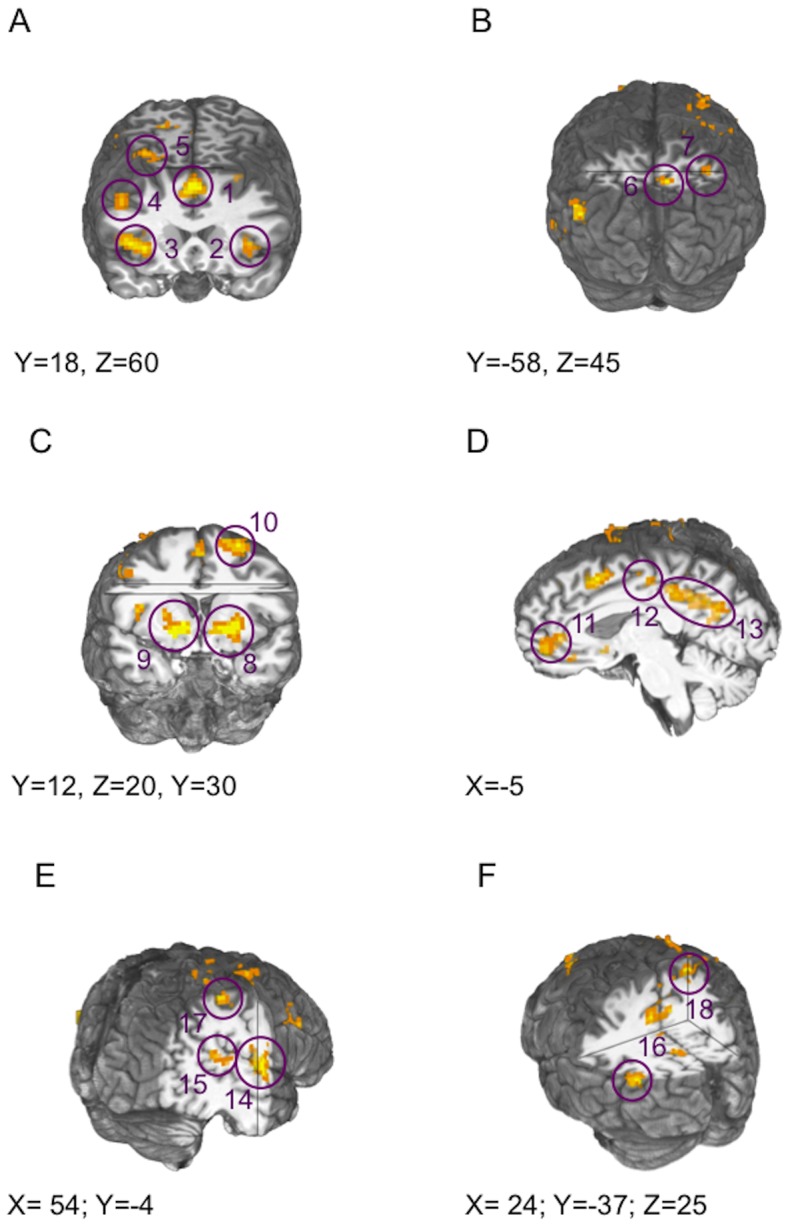
Brain regions showing main effects of FEEDBACK VALENCE on BOLD responses (Talairach coordinates of each slice listed at the bottom of each panel). (**A**) BOLD ACTIVATIONS in response to negative (relative to positive) feedback are evident in insula/vlPFC, dmPFC, dlPFC, and (**B**) posterior parietal cortex, in the entire sample. (**C**) BOLD DEACTIVATIONS in response to negative (relative to positive) feedback are evident in ventral striatum, (**D**) anterior and posterior cingulate cortex, (**E**) lateral temporal cortex, and (**F**) posterior parietal cortex, in the entire sample.

Main effects of feedback VALENCE, in this contrast, were again of two types: those showing greater activation for positive than negative feedback, and those showing the opposite pattern (Table 5). As depicted in Figure 3A–B, areas showing greater activation in response to negative vs. positive feedback included dlPFC, vlPFC, and PPC. In contrast, areas showing greater activity in response to positive vs. negative feedback included the vmPFC and neostriatum (Figure 3C–F), which have been typically associated with the integration of outcomes and the signaling of reward prediction errors [4], [5], [54]. In fact, the large striatal regions showing valence sensitivity in our study encompassed the coordinates of the ventromedial striatal area (±10, 8, −4) reported by Cools et al. [1]. In addition to vmPFC and neostriatum, however, numerous brain areas not strictly linked to outcome processing showed strong deactivations in response to negative, relative to positive feedback. These areas comprise many hypothesized components of the brain's DMN, including structures along the midline, as well as lateral temporal cortex and PPC.

**Table 5:** Brain regions showing main effects of feedback valence: [Lose-Stay-Win-stay] contrast.

Lose-stay > Win-stay										
				x	,	y	,	z		
Brain Region (Cluster #)		BA	L/R	(R+	,	A+	,	S+)	Voxels	Vol (µl)
	*Frontal Cortex*									
1.	dmPFC‡	8		1	,	19	,	46	151	4077
2.	Insula/vlPFC	13	L	−36	,	20	,	1	24	648
3.	Insula/vlPFC‡	13	R	39	,	19	,	3	101	2727
4.	dlPFC‡	8/9	R	47	,	17	,	35	89	2403
5.	SMA	6	R	28	,	0	,	60	71	1917
	*Parietal Cortex*									
6.	Precuneus	7	R	8	,	−71	,	45	25	675
7.	Inferior Parietal Lobule	7	R	35	,	−58	,	46	22	594

Lose-stay<Win-stay										
				x	,	y	,	z		
Brain Region (Cluster #)		BA	L/R	(R+	,	A+	,	S+)	Voxels	Vol (µl)
	*Basal Ganglia*									
8.	Putamen		L	−25	,	−2	,	−5	405	10935
9.	Putamen		R	26	,	−8	,	0	603	16281
										
	*Frontal Cortex*									
10.	Superior Frontal G.	8	L	−21	,	31	,	47	104	2808
11.	vmPFC/Pregenual ACC*	32		−2	,	42	,	−3	116	3132
12.	Motor Cingulate	6	R	3	,	−18	,	48	77	2079
13.	Posterior Cingulate*	31	L	−5	,	−49	,	31	118	3186
	*Temporal/Parietal Cortex*									
14.	STG†	22	R	56	,	−4	,	9	104	2808
15.	TOJ	42	R	53	,	−31	,	13	89	2403
16.	Middle Temporal G.	39	L	−46	,	−73	,	25	58	1566
17.	Postcentral G.	2	R	49	,	−28	,	53	21	567
18.	Postcentral G.	2	R	24	,	−37	,	64	26	702

Brain region numbers correspond to those illustrated in Figure? 2.

* = Overlaps with Default Mode Network component cluster (Table? 2).

† = Overlaps with region showing group by valence interaction (Table? 4).

‡ = Overlaps with region showing main effect of behavior (shift vs. stay; Table? 5).

Abbreviations: BA, Brodmann Area; L, left; R, right; A, anterior; S, superior; Vol (µl), volume in microliters; dmPFC, dorsomedial prefrontal cortex; vlPFC, ventrolateral prefrontal cortex; dlPFC, dorsolateral prefrontal cortex; SMA, supplementary motor area; vmPFC, ventromedial prefrontal cortex; ACC, anterior cingulate cortex; STG, superior temporal gyrus; TOJ, tempero-occipital junction; mid., middle.

### Whole-brain Analyses: Lose-shifts vs. Lose-stays

**Figure 4:**
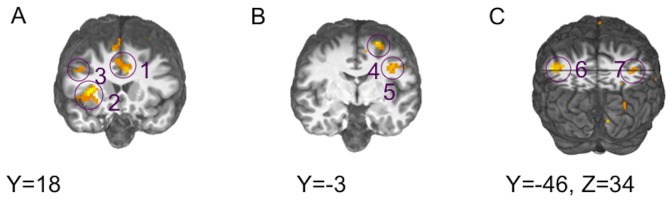
Brain regions showing main effects of FEEDBACK-EVOKED BEHAVIOR (stay vs. shift) on BOLD responses, in the entire sample (Talairach coordinates of each slice listed at the bottom of each panel). Decisions to shift to the alternate stimuli following negative feedback were associated with activations (relatives to stays) in (**A**) dmPFC, R dlPFC, R vlPFC, (**B**) premotor cortex, and (**C**) posterior parietal cortex.

Table 6 lists areas showing main effects of feedback-evoked BEHAVIOR ([Lose-shift-Lose-stay] contrasts). Activated areas included many of the same regions responding to negative feedback, including vlPFC, dlPFC (BA 9), dmPFC, and IPL (Figure 4). No areas showed significant GROUP×BEHAVIOR interactions at the minimal cluster size threshold [an area of dlPFC/frontopolar cortex (Talairach coordinates: −40, 48, 20) showed a GROUP×BEHAVIOR interaction trend at p = 0.001, uncorrected (cluster size: 13 voxels)].

**Table 6:** Brain areas showing greater activations for lose-shifts than lose-stays.

				x	,	y	,	z		
Brain Region (Cluster #)		BA	L/R	(R+	,	A+	,	S+)	Voxels	Vol (µl)
	*Frontal Cortex*									
1.	dmPFC	8		2	,	13	,	46	146	3942
2.	Insula/vlPFC	13	R	36	,	18	,	4	100	2700
3.	dlLPFC	8/9	R	46	,	19	,	31	21	567
4.	SMA	6	L	−24	,	−3	,	57	30	810
5.	Precentral G.	6	L	−39	,	−5	,	34	32	864
	*Parietal/Occip. Ctx.*									
6.	Supramarginal G.	40	L	−42	,	−45	,	37	50	1350
7.	Supramarginal G.	40	R	46	,	−47	,	32	38	1026
	Lingual G.	18	R	10	,	−63	,	2	72	1944
	Lingual G.	18	L	−15	,	−69	,	−3	153	4131
	Middle Occipital G.	19	R	33	,	−78	,	19	58	1566
	Cuneus	17	L	−21	,	−79	,	22	70	1890
	Cuneus	17	R	13	,	−89	,	10	39	1053
	*Cerebellum*									
	Culmen		R	27	,	−51	,	−11	49	1323
	Culmen		L	−32	,	−52	,	−19	36	972
	Uvula		L	−5	,	−72	,	−27	30	810

Brain region numbers correspond to those illustrated in Figure? 4.

Abbreviations: BA, Brodmann Area; L, left; R, right; A, anterior; S, superior; Vol (µl), volume in microliters; dmPFC, dorsomedial prefrontal cortex; vlPFC, ventrolateral prefrontal cortex; dlPFC, dorsolateral prefrontal cortex; SMA, Supplementary Motor Area; G., gyrus; mid., middle.

### Regions-of-interest Analyses: Effects of group and feedback-type

**Figure 5:**
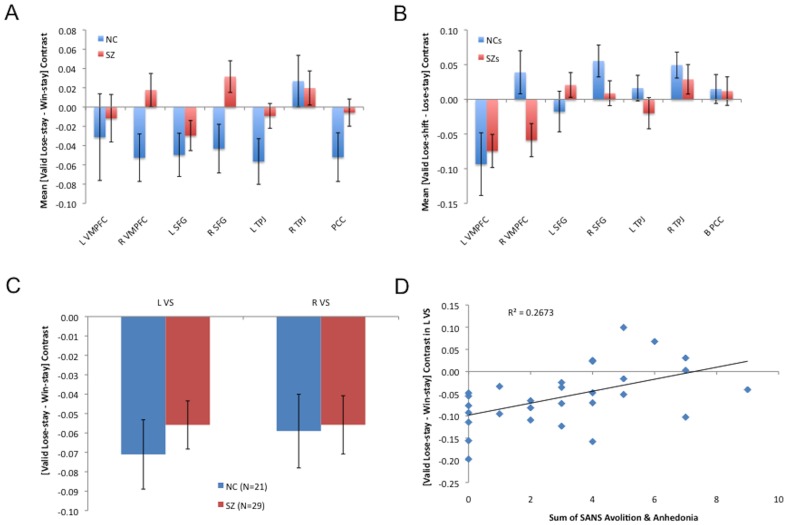
Responses in patients and controls in ROIs in default mode and reward networks. (**A**) Responses to lose-stays (relative to win-stays) in default network nodes. Patients showed reduced BOLD signal contrasts between valid lose-stays and valid win-stays in two components of default networks in frontal cortex. (**B**) Responses to lose-shifts (relative to lose-stays) in default network nodes in default network nodes. Patients showed deactivations for lose-shifts, relative to lose-stays in right mPFC, whereas controls showed the opposite pattern. (**C**) Responses to negative feedback (relative to positive feedback) in left and right ventral striatum (±10, 8, −4). Both patients and controls strongly deactivated left and right VS for lose-stays, relative to win-stays. (**D**) Deactivation of left VS, in SZ patients, in response to lose-stays (relative to win-stays) correlated significantly with clinical ratings of avolition/anhedonia.

In order to examine more closely effects of GROUP on feedback-related responses, we analyzed the effects of feedback-valence in ventral striatal ROIs and within components of the DMN, identified through an independent data set and rsFC. Analyses of variance revealed no significant GROUP×VALENCE interactions in either left or right VS, but showed main effects in both areas (Figure 5C, Table S1). In DMN ROIs, analyses of variance revealed significant GROUP×VALENCE interactions in right mPFC and right SFG (with trends toward significant interaction in PCC and left PPC; Figure 5A, Table S1). Thus, while whole-brain analyses did not reveal any group differences in the magnitudes of their neural responses in reward network components, ROI analyses indicated that infrequent negative and frequent positive feedback instances evoked differential responses in multiple DMN nodes in the patients and controls.

Analyses of variance revealed no significant GROUP×BEHAVIOR (lose-shift vs. lose-stay) interactions in left or right VS, and no main effects in either area (Table S3). In DMN ROIs, analyses of variance revealed a significant GROUP×BEHAVIOR interaction in right mPFC (Figure 5B, Table S3). Main effects of loss-evoked behavior were observed in left mPFC, right SFG, and right PPC. Main effects of valence were observed in PCC, left PPC, and left SFG. Thus, ROI analyses indicated that pre-reversal errors evoked, in controls, greater deactivations of right mPFC than final reversal errors, whereas SZ patients showed the opposite pattern.

### Correlation Analyses in ROIs

Ratings for anhedonia/avolition correlated significantly with valence contrasts ([valid negative feedback-valid positive feedback]) in two reward network nodes: vmPFC and left putamen/ventral striatum (Figure 5D, Table S5). Ratings for anhedonia/avolition correlated significantly with evoked-behavior contrasts ([lose-shift-lose-stay]) in left Brodmann Area 6 (Table S6). No other significant correlations among clinical symptom ratings and shift-evoked neural activity were observed. These results provide evidence that reduced relative deactivation of reward network components in the face of surprising negative feedback may figure in the motivational deficits in SZ patients, in particular.

Haloperidol-equivalent antipsychotic doses correlated significantly with valence contrasts in several DMN nodes: right mPFC, right PPC, and left SFG (Table S7). No significant correlations were observed among haloperidol-equivalent antipsychotic doses and valence contrasts in VS or vmPFC, and no significant correlations were observed among antipsychotic doses and shift-evoked neural activity in DMN nodes. These findings indicate that the correlations between MRI signal contrasts in reward circuit nodes and clinical symptoms, observed in patients, were not attributable to antipsychotic medication status.

## Discussion

In this MRI study of probabilistic reversal learning in chronic, medicated patients with schizophrenia and matched controls, we observed, as predicted, that measures of striatal sensitivity to outcome valence did not differ between controls and the entire sample of patients, but *did* correlate with ratings of negative symptoms *within* the patient group. Also consistent with our hypothesis, we observed that SZ patients showed abnormal responses to feedback in several default-mode regions (mPFC and superior frontal gyrus). Our hypothesis that SZ patients would show abnormal neural correlates of choices to switch following negative feedback in components of an executive control network was only weakly supported by the results of our whole-brain analysis. Consistent with our previous work, SZ patients made increased numbers of errors, and achieved fewer stages, in performing the PRL task. However, performance deficits in patients appeared to be driven by excessive rates of shifting in response to instances of positive and invalid negative feedback, rather than being attributable to an increased tendency to perseverate (to make the same response in response to valid negative feedback).

### Striatal responses to negative feedback in SZ patients correlate with negative-symptom severity

Importantly, when we looked specifically in striatum, we found no group differences in valence sensitivity, but, consistent with previous findings from our group and other groups, we observed significant correlations between neural responses and ratings of negative symptoms in patients. For example, correlations have been observed between negative symptom ratings and striatal activity associated with both anticipation of a symbolic reward (money) [16], [55] and the receipt of a primary reinforcer [56]. In the present study we found that sensitivity to the valence of a monetary outcome in the left striatum, manifest as the ability to deactivate striatum in response to surprising losses, correlated with clinical ratings for avolition/anhedonia. This suggests that, in SZ patients with severe negative symptoms, sensitivity to outcome valence may also be attenuated and that striatal responses to reinforcers are a factor in motivational deficits.

### Abnormal feedback-related activity in DMN components

Reduced deactivations of mPFC and SFG following salient negative feedback were characteristic of SZ patients, as a group. Our finding of a reduced ability to suppress DMN activity in patients with SZ is consistent with a growing body of evidence [26], [27], [57]. Building on previous evidence of a failure to suppress DMN activity during the completion of attention-demanding cognitive tasks in SZ patients [26], the current findings show that a failure to suppress default network activity may also contribute to deficits in reinforcement learning observed in SZ patients.

### Punishment-driven set-shifting and neural networks for executive control and attention

Consistent with numerous previous studies [6], [58], [59], negative feedback activated a set of brain areas in our study that included bilateral insula/vlPFC, dlPFC, dmPFC, lateral premotor cortex, and PPC. Further, analyses of neural activity predictive of successful reversals (valid negative feedback leading to shifts rather than stays) revealed a set of regions (including dmPFC, dlPFC, insula/vlPFC, supplementary motor area, lateral premotor cortex, PPC, and cerebellum) that was also largely consistent with previous work. Importantly, dmPFC and vlPFC have been specifically implicated in response inhibition and set-shifting [60]–[62]. In fact, the centers-of-mass coordinates of the dmPFC (2, 13, 46) and vlPFC (36, 18, 4) regions we report as showing main effects of shifting (as opposed to staying) were close to those reported by Cools and colleagues [1] (dmPFC: 8, 32, 52; 10, 10, 52; vlPFC: 38, 24, −2), who performed the study of probabilistic reversal learning upon which the current one was based.

Based on our observations of group differences in behavioral measures related to response-set-shifting behavior, we expected to find significant differences between SZ patients and controls in signal contrasts characterizing shift-evoked activity. Although our study was perhaps underpowered to find such group differences, a small cluster in dlPFC/frontopolar cortex emerged from the whole-brain analysis at the uncorrected voxel-wise threshold (p = 0.001), suggesting possible group differences in the physiology of response-set-shifting. Based on the current results, further investigations into the disruption or intactness of response inhibition and set-shifting signals in SZ patients, in the context of reinforcement learning, are warranted.

It is noteworthy that significant differences emerged between SZ patients and controls in BOLD signal contrasts characterizing shift-evoked activity in two parietal, hypothesized dorsal stream regions: inferior parietal lobule (BA 40) and precuneus. Interestingly, functions beyond purely visual/spatial processing have been ascribed to these regions. Inferior parietal lobule, for example, has been linked to “maintaining attentive control on current task goals as well as responding to salient new information or alerting stimuli in the environment” [63], whereas the precuneus has been associated with self-referential processing, especially at rest [64]. These functions relate closely to the concept of executive control, and, not surprisingly, extensive connections exist between these areas and executive control nodes in the frontal cortex [64], [65].

### Excessive shifting in schizophrenia patients

Whereas previous findings have pointed to perseveration in SZ patients [66], [67], our results indicate that patients switch in response to valid instances of negative feedback at a rate comparable to controls. However, patients' rates of maladaptively shifting to *other* kinds of feedback (e.g., rewards and probabilistic errors) exceed those of controls, and thus SZ patients switch less-*selectively* to valid instances of negative feedback, accounting for their lower average numbers of stages achieved. This finding suggests that, rather than being perseverative in their behavior, SZ patients often have difficulty acquiring learning sets *in the first place*. This behavioral finding is consistent with our MRI findings that SZ patients show normal activations of vlPFC, dlPFC, and dmPFC in response to valid negative feedback, but *larger than normal* deactivations of vmPFC to *positive* feedback.

### Limitations of the study

The interpretations of this study are potentially limited by several factors. First, because subjects were trained prior to scanning to do the PRL task, and because the worst-performing (and, likely, most intellectually impaired) subjects were excluded from analyses, due to the low numbers of events of interest, our results do not consider cases where difficulty in acquiring and reversing discriminations was the greatest. Training was done in order to minimize frustration in subjects, and because one of our main foci was on neural activity patterns distinguishing successful reversals from failed reversals. Second, it could be argued that attenuated contrasts in SZ patients reflect reduced engagement in the task. While we cannot rule out differences in behavioral performance as contributors to differences in neural activity, it should be noted that no group differences were observed in punishment-evoked *activations*; that is, many neural responses, including feedback-evoked responses in some brain areas, were found to be normal in patients. Furthermore, we did not contrast event types associated with varying numbers of correct responses: we contrasted responses evoked by valid and invalid rewards and punishments, regardless of how many events of each type there were in individual subjects. Third, though the patterns of group differences in neural activity point to deficits in attentional set-shifting in SZ patients, the current study lacked a direct manipulation of the information-processing/attentional demands of a shift (for example, in the manner of [68]). Future studies of PRL/rapid reinforcement learning in SZ might include such a manipulation, in order to directly examine this issue. Finally, the design of our task, with the choice presentation followed closely by the response and feedback, prevented the use of the choice presentation, response, and feedback events as independent regressors. To overcome this limitation, we identified response feedback sequences by their valence, validity, and ensuing choice, and performed second-level analyses by contrasting trials of differing valence, holding constant their validity and the ensuing behavior (e.g., a stay), or by contrasting feedback events leading to different choices (shift or stay) holding constant their valence and validity. Future studies of PRL in SZ might involve greater separation in time of choice, response, and feedback events.

Another potential confound is the fact that all of our subjects were outpatients stably-treated with antipsychotic medications, and, thus, we considered the possible impact of dopamine receptor blockade on reward-related neural responses. To determine whether group differences were attributable to the medication, rather than illness, we computed correlations between measures of brain activity and haloperidol-equivalent doses of antipsychotic drugs [51]. We observed no significant correlations between haloperidol equivalent antipsychotic drug dose and any of the neural signals showing systematic relationships with negative symptom ratings (in VS and vmPFC; see Tables S5, S6, S7, S8). Therefore, there is little indication that antipsychotic medication status drove the observed effects of negative symptom severity; it is more likely that symptom severity drove the effect of antipsychotic use. That is, any significant correlation between reward-related brain responses and antipsychotic dose would likely be secondary to the fact that the most symptomatic patients in our study were taking the highest doses of antipsychotic medication.

Finally, because schizophrenia disproportionately affects males [69], and because men make up roughly 65% of the population of MPRC outpatient clinics (the source of our patient sample), males and females were not equally represented in our sample. Because of this unequal gender distribution, our results may not generalize completely to the population at large.

### General implications: Multiple processes in reinforcement learning are disrupted in SZ patients with motivational deficits

Our behavioral and MRI data suggest that PRL deficits in most SZ patients likely do not simply result from a simple insensitivity to negative feedback. Rather, we provide evidence that motivational deficits in schizophrenia travel with aspects of abnormal processing in three relatively distinct, functionally-defined brain circuits: 1) a network of reward processing structures, 2) a default mode network, and 3) an executive control network. In other words, an individual with SZ could show disruption in any or all of these circuits, and show poor reversal learning performance as a consequence of dysfunction in any combination of these circuits. The fact that successful reversal learning depends on the ability of these circuits to work in concert further suggests that faulty *interactions among* circuits and networks could be at the root of poor reversal learning performance in SZ patients-especially those with severe negative symptoms.

Given the purported role of DMN suppression in task-focused attention [20], [23]–[25], DMN deactivations to valid instances of negative feedback evoking stays may reflect adaptive enhancements of attention to ensuing choice-feedback sequences. Importantly, even if unexpected instances of negative feedback are salient enough to result in largely normal negative prediction error signals in the striatum in SZ patients, several factors may interfere with the acquisition and maintenance of new sets in SZ: 1) unexpected instances of negative feedback may not lead to adaptive suppressions of DMN activity, and 2) instances of *positive* feedback may be excessively salient, leading to exaggerated suppressions of DMN activity, making reversal signals harder to detect. Recent theoretical approaches [18], [19] suggest that a salience network, including ACC and anterior insula, make a critical contribution to the ability to switch between executive control and default mode brain networks. The observation that negative symptoms in SZ traveled with neural responses in components of both reward and default networks suggests that deficits in motivation exhibited by patients might not just emerge from problems of appropriately integrating outcomes or selecting actions, but rather from difficulty in coordinating processing in multiple, relatively distinct, neural networks.

In conclusion, our results provide support for the idea that patients with SZ show impaired PRL performance because they fail to adaptively modulate attention in response to salient instances of feedback. However, future studies need to investigate this issue directly by systematically manipulating uncertainty/information-processing load in the context of reinforcement learning tasks. Given the prominent role attributed to dorsal ACC in representing the value of actions [12], [70], changes in the strength of stimulus-response-reward associations are likely to be reflected in activity patterns in that region. Given that numerous studies have pointed to abnormal dACC activity in SZ associated with the performance of tasks reliant on cognitive control, future studies might attempt to integrate these findings by determining the impact of the certainty of value representations on reinforcement learning performance in SZ and associated neural signals. Such a manipulation would help to link findings related to deficits in the top-down control of attention in SZ, and impairments in the ability to learn from feedback.

## Supporting Information

Figure S1 **Results of rsFC analyses.** (**A**) Results of rsFC analyses done separately for controls. Panel A shows connectivity between medial prefrontal and posterior cingulate cortex (PCC; cut at x = 3), whereas (**B**) shows connectivity between PCC seed, posterior parietal cortex and superior frontal gyrus (both bilaterally; cut at z = 42). (**C**) Results of rsFC analyses done separately for SZ patients. Panel A shows extended PCC region exhibiting significant connectivity with the seed regions (cut at x = 3). (**D**) SZ patients show significant connectivity between the PCC seed, posterior parietal cortex and superior frontal gyrus (both bilaterally; cut at z = 42). (**E**) Results of rsFC analyses done for the entire sample. Panel E shows connectivity between medial prefrontal and posterior cingulate cortex (PCC; cut at x = 3), whereas (**F**) shows connectivity between PCC seed, posterior parietal cortex and superior frontal gyrus (both bilaterally; cut at z = 42).
(TIF)

Table S1 Results of ANOVAs examining feedback-evoked deactivations in default mode network ROIs, with factors of GROUP (patients vs. controls) and FEEDBACK-VALENCE (negative vs. positive). Analyses of variance revealed no significant GROUP×VALENCE interactions in either left or right VS, but showed main effects in both areas. In DMN ROIs, analyses of variance revealed significant GROUP×VALENCE interactions in right mPFC and right SFG (with trends toward significant interaction in PCC and left PPC).
(DOC)

Table S2 Results of ANOVAs examining feedback-evoked deactivations in DMN ROIs, with factors of GROUP (patients vs. controls) and FEEDBACK-VALENCE (negative vs. positive): Comparisons of cell-means. Patients and controls showed significantly different responses to negative feedback in L PPC and to positive feedback in R SFG.
(DOC)

Table S3 Results of ANOVAs examining feedback-evoked deactivations in default mode network ROIs, with factors of GROUP (patients vs. controls) and BEHAVIOR (lose-shift vs. lose-stay). Analyses of variance revealed no significant GROUP×BEHAVIOR (lose-shift vs. lose-stay) interactions in left or right VS, and no main effects in either area (Table S3). In DMN ROIs, analyses of variance revealed a significant GROUP×BEHAVIOR interaction in right mPFC. Main effects of loss-evoked behavior were observed in left mPFC, right SFG, and right PPC. Main effects of valence were observed in PCC, left PPC, and left SFG.
(DOC)

Table S4 Results of ANOVAs examining behavior-evoked deactivations in DMN ROIs, with factors of GROUP (patients vs. controls) and EVOKED-BEHAVIOR (lose-shift vs. win-stay): Comparisons of cell-means. Patients and controls showed significantly different responses to lose-shifts in L SFG and to lose-stays in L PPC.
(DOC)

Table S5 Correlations between average Avolition/Anhedonia ratings and valence contrasts ([valid negative feedback-valid positive feedback]) in Network Components. Ratings for anhedonia/avolition correlated significantly with valence contrasts in two reward network nodes: vmPFC and left putamen/ventral striatum.
(DOC)

Table S6 Correlations between average Avolition/Anhedonia ratings and behavior ([Lose-stay-Lose-shift]) contrasts in Network Components. Ratings for anhedonia/avolition correlated significantly with evoked-behavior contrasts ([lose-shift-lose-stay]) in left Brodmann Area 6. No other significant correlations among clinical symptom ratings and shift-evoked neural activity were observed.
(DOC)

Table S7 Correlations between haloperidol-equivalent antipsychotic dose and valence contrasts in Network Components. Haloperidol-equivalent antipsychotic doses correlated significantly with valence contrasts in several DMN nodes: right mPFC, right PPC, and left SFG. No significant correlations were observed among haloperidol-equivalent antipsychotic doses and valence contrasts in VS or vmPFC.
(DOC)

Table S8 Correlations between haloperidol-equivalent antipsychotic dose and behavior ([Lose-stay-Lose-shift]) contrasts in Network Components. No significant correlations were observed among antipsychotic doses and shift-evoked neural activity in DMN nodes or ECN nodes.
(DOC)

Text S1 Supporting text and references.
(DOC)
