# Supplementary figures and images for: The Roles of Reward, Default, and Executive Control Networks in Set-Shifting Impairments in Schizophrenia
Source: PLoS One. 2013 Feb 27;8(2):e57257. doi: 10.1371/journal.pone.0057257 (PMC3584128; doi:10.1371/journal.pone.0057257)

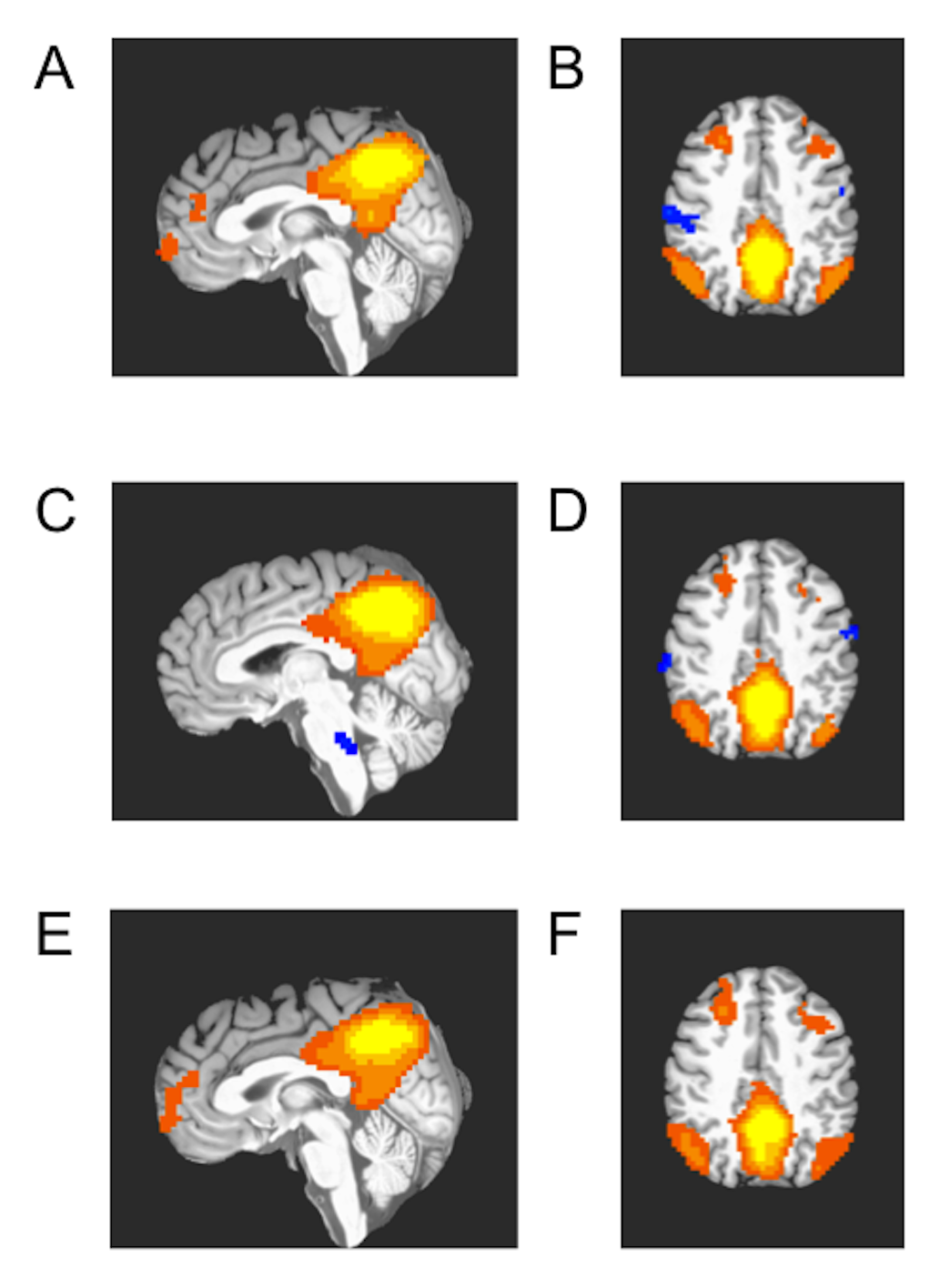

Supplement: Figure S1 — Results of rsFC analyses. (A) Results of rsFC analyses done separately for controls. Panel A shows connectivity between medial prefrontal and posterior cingulate cortex (PCC; cut at x = 3), whereas (B) shows connectivity between PCC seed, posterior parietal cortex and superior frontal gyrus (both bilaterally; cut at z = 42). (C) Results of rsFC analyses done separately for SZ patients. Panel A shows extended PCC region exhibiting significant connectivity with the seed regions (cut at x = 3). (D) SZ patients show significant connectivity between the PCC seed, posterior parietal cortex and superior frontal gyrus (both bilaterally; cut at z = 42). (E) Results of rsFC analyses done for the entire sample. Panel E shows connectivity between medial prefrontal and posterior cingulate cortex (PCC; cut at x = 3), whereas (F) shows connectivity between PCC seed, posterior parietal cortex and superior frontal gyrus (both bilaterally; cut at z = 42). (TIF) [file pone.0057257.s001.tif]
