# Supplementary material for: The Roles of Reward, Default, and Executive Control Networks in Set-Shifting Impairments in Schizophrenia
Source: PLoS One. 2013 Feb 27;8(2):e57257. doi: 10.1371/journal.pone.0057257 (PMC3584128; doi:10.1371/journal.pone.0057257)
Supplement: Table S1 — Results of ANOVAs examining feedback-evoked deactivations in default mode network ROIs, with factors of GROUP (patients vs. controls) and FEEDBACK-VALENCE (negative vs. positive). Analyses of variance revealed no significant GROUP×VALENCE interactions in either left or right VS, but showed main effects in both areas. In DMN ROIs, analyses of variance revealed significant GROUP×VALENCE interactions in right mPFC and right SFG (with trends toward significant interaction in PCC and left PPC). (DOC) [file pone.0057257.s002.doc]

**Table S1. Results of ANOVAs examining feedback-evoked deactivations in DMN ROIs, with factors of GROUP (patients vs. controls) and FEEDBACK-VALENCE (negative vs. positive): Main Effects and Interactions.**

|  | **GROUP x VALENCE**  **Interaction** | |  | **GROUP**  **Main Effect** | |  | **VALENCE**  **Main Effect** | |
| --- | --- | --- | --- | --- | --- | --- | --- | --- |
|  |  |  |  |  |  |  |  |  |
| **ROI** | **F** | **p** |  | **F** | **p** |  | **F** | **p** |
| L VS | 0.521 | 0.474 |  | 0.419 | 0.520 |  | **36.354** | **<0.001** |
| R VS | 0.018 | 0.894 |  | 0.645 | 0.426 |  | **23.075** | **<0.001** |
| L mPFC | 0.167 | 0.684 |  | 0.342 | 0.561 |  | 0.798 | 0.376 |
| R mPFC | **5.836** | **0.020** |  | 0.162 | 0.689 |  | 1.442 | 0.236 |
| L SFG | 0.574 | 0.452 |  | 0.834 | 0.366 |  | **8.967** | **0.004** |
| R SFG | **6.719** | **0.013** |  | 0.870 | 0.356 |  | 0.161 | 0.690 |
| L PPC | *3.552* | *0.066* |  | *3.430* | *0.070* |  | **6.835** | **0.012** |
| R PPC | 0.054 | 0.817 |  | 0.492 | 0.486 |  | 2.296 | 0.136 |
| PCC | *2.906* | *0.095* |  | 0.837 | 0.365 |  | **4.543** | **0.038** |

Abbreviations: ROI, region of interest; R, right; VS, ventral striatum; L, left; vmPFC, ventromedial prefrontal cortex; ITG, inferior temporal gyrus; PHG, parahippocampal gyrus; PCC, posterior cingulate cortex; DMPFC, dorsomedial prefrontal cortex; DLPFC, dorsolateral prefrontal cortex; BA6, Brodmann Area 6.
