# Supplementary material for: The Roles of Reward, Default, and Executive Control Networks in Set-Shifting Impairments in Schizophrenia
Source: PLoS One. 2013 Feb 27;8(2):e57257. doi: 10.1371/journal.pone.0057257 (PMC3584128; doi:10.1371/journal.pone.0057257)
Supplement: Table S2 — Results of ANOVAs examining feedback-evoked deactivations in DMN ROIs, with factors of GROUP (patients vs. controls) and FEEDBACK-VALENCE (negative vs. positive): Comparisons of cell-means. Patients and controls showed significantly different responses to negative feedback in L PPC and to positive feedback in R SFG. (DOC) [file pone.0057257.s003.doc]

**Table S2. Results of ANOVAs examining feedback-evoked deactivations in DMN ROIs, with factors of GROUP (patients vs. controls) and FEEDBACK-VALENCE (negative vs. positive): Comparisons of cell-means.**

|  | **Valid Negative**  **Feedback** | |  | **Valid Positive**  **Feedback** | |
| --- | --- | --- | --- | --- | --- |
|  |  |  |  |  |  |
| **ROI** | **t** | **p** |  | **t** | **p** |
| L VS | 0.393 | 0.696 |  | 0.880 | 0.383 |
| R VS | 0.681 | 0.499 |  | 0.826 | 0.413 |
| L mPFC | 0.332 | 0.742 |  | 0.853 | 0.398 |
| R mPFC | -0.449 | 0.655 |  | 1.327 | 0.191 |
| L SFG | -1.089 | 0.282 |  | -0.548 | 0.586 |
| R SFG | -0.242 | 0.810 |  | **2.466** | **0.017** |
| L PPC | **-2.238** | **0.030** |  | -1.099 | 0.277 |
| R PPC | -0.467 | 0.643 |  | -0.877 | 0.385 |
| PCC | -1.416 | 0.163 |  | -0.176 | 0.861 |

Abbreviations: ROI, region of interest; R, right; VS, ventral striatum; L, left; vmPFC, ventromedial prefrontal cortex; ITG, inferior temporal gyrus; PHG, parahippocampal gyrus; PCC, posterior cingulate cortex; DMPFC, dorsomedial prefrontal cortex; DLPFC, dorsolateral prefrontal cortex; BA6, Brodmann Area 6.
