# Supplementary material for: The Roles of Reward, Default, and Executive Control Networks in Set-Shifting Impairments in Schizophrenia
Source: PLoS One. 2013 Feb 27;8(2):e57257. doi: 10.1371/journal.pone.0057257 (PMC3584128; doi:10.1371/journal.pone.0057257)
Supplement: Table S3 — Results of ANOVAs examining feedback-evoked deactivations in default mode network ROIs, with factors of GROUP (patients vs. controls) and BEHAVIOR (lose-shift vs. lose-stay). Analyses of variance revealed no significant GROUP×BEHAVIOR (lose-shift vs. lose-stay) interactions in left or right VS, and no main effects in either area (Table S3). In DMN ROIs, analyses of variance revealed a significant GROUP×BEHAVIOR interaction in right mPFC. Main effects of loss-evoked behavior were observed in left mPFC, right SFG, and right PPC. Main effects of valence were observed in PCC, left PPC, and left SFG. (DOC) [file pone.0057257.s004.doc]

**Table S3. Results of ANOVAs examining behavior-evoked deactivations in DMN ROIs, with factors of GROUP (patients vs. controls) and EVOKED-BEHAVIOR (lose-shift vs. win-stay): Main Effects and Interactions.**

|  | **GROUP x BEHAV**  **Interaction** | |  | **GROUP**  **Main Effect** | |  | **BEHAVIOR**  **Main Effect** | |
| --- | --- | --- | --- | --- | --- | --- | --- | --- |
|  |  |  |  |  |  |  |  |  |
| **ROI** | **F** | **p** |  | **F** | **p** |  | **F** | **p** |
| **L VS** | 1.809 | 0.185 |  | 1.040 | 0.313 |  | 0.532 | 0.470 |
| **R VS** | 0.026 | 0.872 |  | 0.812 | 0.372 |  | 0.000 | 0.984 |
| **L mPFC** | -0.399 | 0.692 |  | 0.062 | 0.804 |  | **12.487** | **0.001** |
| **R mPFC** | **2.538** | **0.014** |  | 0.603 | 0.441 |  | 0.267 | 0.608 |
| **L SFG** | -1.179 | 0.244 |  | *3.656* | *0.062* |  | 0.010 | 0.922 |
| **R SFG** | 1.618 | 0.112 |  | 0.173 | 0.679 |  | **4.979** | **0.030** |
| **L PPC** | 1.175 | 0.246 |  | *3.919* | *0.053* |  | 0.015 | 0.904 |
| **R PPC** | 0.695 | 0.490 |  | 0.061 | 0.806 |  | **6.986** | **0.011** |
| **PCC** | 0.101 | 0.920 |  | 2.682 | 0.108 |  | 0.798 | 0.376 |

Abbreviations: ROI, region of interest; R, right; VS, ventral striatum; L, left; vmPFC, ventromedial prefrontal cortex; ITG, inferior temporal gyrus; PHG, parahippocampal gyrus; PCC, posterior cingulate cortex; DMPFC, dorsomedial prefrontal cortex; DLPFC, dorsolateral prefrontal cortex; BA6, Brodmann Area 6.
