# Supplementary material for: The Roles of Reward, Default, and Executive Control Networks in Set-Shifting Impairments in Schizophrenia
Source: PLoS One. 2013 Feb 27;8(2):e57257. doi: 10.1371/journal.pone.0057257 (PMC3584128; doi:10.1371/journal.pone.0057257)
Supplement: Table S4 — Results of ANOVAs examining behavior-evoked deactivations in DMN ROIs, with factors of GROUP (patients vs. controls) and EVOKED-BEHAVIOR (lose-shift vs. win-stay): Comparisons of cell-means. Patients and controls showed significantly different responses to lose-shifts in L SFG and to lose-stays in L PPC. (DOC) [file pone.0057257.s005.doc]

**Table S4. Results of ANOVAs examining behavior-evoked deactivations in DMN ROIs, with factors of GROUP (patients vs. controls) and EVOKED-BEHAVIOR (lose-shift vs. win-stay): Comparisons of cell-means.**

|  | **Valid**  **Lose-shift** | |  | **Valid**  **Lose-stay** | |
| --- | --- | --- | --- | --- | --- |
|  |  |  |  |  |  |
| **ROI** | **t** | **p** |  | **t** | **p** |
| L VS | 1.631 | 0.109 |  | 0.393 | 0.696 |
| R VS | 0.951 | 0.346 |  | 0.681 | 0.499 |
| L mPFC | 0.096 | 0.924 |  | 0.332 | 0.742 |
| R mPFC | *1.922* | *0.061* |  | -0.449 | 0.655 |
| L SFG | **-2.261** | **0.028** |  | -1.089 | 0.282 |
| R SFG | 1.114 | 0.271 |  | -0.242 | 0.810 |
| L PPC | -1.294 | 0.202 |  | **-2.238** | **0.030** |
| R PPC | 0.046 | 0.964 |  | -0.467 | 0.643 |
| PCC | -1.543 | 0.129 |  | -1.416 | 0.163 |

Abbreviations: ROI, region of interest; R, right; VS, ventral striatum; L, left; vmPFC, ventromedial prefrontal cortex; ITG, inferior temporal gyrus; PHG, parahippocampal gyrus; PCC, posterior cingulate cortex; DMPFC, dorsomedial prefrontal cortex; DLPFC, dorsolateral prefrontal cortex; BA6, Brodmann Area 6.
