# Supplementary material for: The Roles of Reward, Default, and Executive Control Networks in Set-Shifting Impairments in Schizophrenia
Source: PLoS One. 2013 Feb 27;8(2):e57257. doi: 10.1371/journal.pone.0057257 (PMC3584128; doi:10.1371/journal.pone.0057257)
Supplement: Table S5 — Correlations between average Avolition/Anhedonia ratings and valence contrasts ([valid negative feedback-valid positive feedback]) in Network Components. Ratings for anhedonia/avolition correlated significantly with valence contrasts in two reward network nodes: vmPFC and left putamen/ventral striatum. (DOC) [file pone.0057257.s006.doc]

**Table S5. Correlations between average Avolition/Anhedonia ratings and valence contrasts in Network Components**

| **ROI** | |  | **r** |  | |  | **p** |  |
| --- | --- | --- | --- | --- | --- | --- | --- | --- |
| ***Reward ROIs*** | | | | | | | | |
|  | **L VS** | **0.517** | | | **0.004** | | | |
|  | R VS | 0.283 | | | 0.136 | | | |
|  | **vmPFC** | **0.481** | | | **0.008** | | | |
|  |  |  | | |  | | | |
| ***Default Network*** | | | | | | | | |
|  | L mPFC | 0.107 | | | 0.580 | | | |
|  | *R mPFC* | *0.343* | | | *0.068* | | | |
|  | L SFG | -0.229 | | | 0.232 | | | |
|  | *R SFG* | *0.313* | | | *0.099* | | | |
|  | *L TPJ* | *0.320* | | | *0.091* | | | |
|  | R TPJ | 0.242 | | | 0.207 | | | |
|  | *PCC* | *0.311* | | | *0.100* | | | |

Abbreviations: ROI, region of interest; R, right; VS, ventral striatum; L, left; vmPFC, ventromedial prefrontal cortex; ITG, inferior temporal gyrus; PHG, parahippocampal gyrus; PCC, posterior cingulate cortex; DMPFC, dorsomedial prefrontal cortex; DLPFC, dorsolateral prefrontal cortex; BA6, Brodmann Area 6.
