# Supplementary material for: The Roles of Reward, Default, and Executive Control Networks in Set-Shifting Impairments in Schizophrenia
Source: PLoS One. 2013 Feb 27;8(2):e57257. doi: 10.1371/journal.pone.0057257 (PMC3584128; doi:10.1371/journal.pone.0057257)
Supplement: Table S6 — Correlations between average Avolition/Anhedonia ratings and behavior ([Lose-stay-Lose-shift]) contrasts in Network Components. Ratings for anhedonia/avolition correlated significantly with evoked-behavior contrasts ([lose-shift-lose-stay]) in left Brodmann Area 6. No other significant correlations among clinical symptom ratings and shift-evoked neural activity were observed. (DOC) [file pone.0057257.s007.doc]

**Table S6. Correlations between average Avolition/Anhedonia ratings and behavior ([Lose-stay – Lose-shift]) contrasts in Network Components**

| **ROI** | |  | **r** |  | |  | **p** |  |
| --- | --- | --- | --- | --- | --- | --- | --- | --- |
| ***Executive Control Network*** | | | | | | | | |
|  | DMPFC | -0.058 | | | 0.766 | | | |
|  | R VLPFC | 0.105 | | | 0.587 | | | |
|  | R DLPFC | 0.060 | | | 0.758 | | | |
|  | L BA6 1 | 0.041 | | | 0.832 | | | |
|  | **L BA6 2** | **0.373** | | | **0.046** | | | |
|  |  |  | | |  | | | |
| ***Default Network*** | | | | | | | | |
|  | **L mPFC** | 0.047 | | | 0.807 | | | |
|  | R mPFC | -0.172 | | | 0.371 | | | |
|  | L SFG | 0.277 | | | 0.146 | | | |
|  | R SFG | -0.269 | | | 0.158 | | | |
|  | L TPJ | -0.265 | | | 0.164 | | | |
|  | R TPJ | -0.232 | | | 0.227 | | | |
|  | PCC | -0.198 | | | 0.302 | | | |

Abbreviations: ROI, region of interest; R, right; VS, ventral striatum; L, left; vmPFC, ventromedial prefrontal cortex; ITG, inferior temporal gyrus; PHG, parahippocampal gyrus; PCC, posterior cingulate cortex; DMPFC, dorsomedial prefrontal cortex; DLPFC, dorsolateral prefrontal cortex; BA6, Brodmann Area 6.
