# Supplementary material for: The Roles of Reward, Default, and Executive Control Networks in Set-Shifting Impairments in Schizophrenia
Source: PLoS One. 2013 Feb 27;8(2):e57257. doi: 10.1371/journal.pone.0057257 (PMC3584128; doi:10.1371/journal.pone.0057257)
Supplement: Table S7 — Correlations between haloperidol-equivalent antipsychotic dose and valence contrasts in Network Components. Haloperidol-equivalent antipsychotic doses correlated significantly with valence contrasts in several DMN nodes: right mPFC, right PPC, and left SFG. No significant correlations were observed among haloperidol-equivalent antipsychotic doses and valence contrasts in VS or vmPFC. (DOC) [file pone.0057257.s008.doc]

**Table S7. Correlations between haloperidol-equivalent antipsychotic dose and valence contrasts in Network Components**

| **ROI** | |  | **r** |  | |  | **p** |  |
| --- | --- | --- | --- | --- | --- | --- | --- | --- |
| ***Reward ROIs*** | | | | | | | | |
|  | **L VS** | -0.259 | | | 0.176 | | | |
|  | R VS | -0.077 | | | 0.693 | | | |
|  | **vmPFC** | -0.145 | | | 0.450 | | | |
|  |  |  | | |  | | | |
| ***Default Network*** | | | | | | | | |
|  | *L mPFC* | *-0.338* | | | *0.073* | | | |
|  | **R mPFC** | **-0.418** | | | **0.024** | | | |
|  | **L SFG** | **-0.433** | | | **0.019** | | | |
|  | R SFG | 0.086 | | | 0.659 | | | |
|  | L TPJ | -0.268 | | | 0.160 | | | |
|  | **R TPJ** | **-0.378** | | | **0.043** | | | |
|  | PCC | 0.016 | | | 0.934 | | | |

Abbreviations: ROI, region of interest; R, right; VS, ventral striatum; L, left; vmPFC, ventromedial prefrontal cortex; ITG, inferior temporal gyrus; PHG, parahippocampal gyrus; PCC, posterior cingulate cortex; DMPFC, dorsomedial prefrontal cortex; DLPFC, dorsolateral prefrontal cortex; BA6, Brodmann Area 6.
