# Supplementary material for: The Roles of Reward, Default, and Executive Control Networks in Set-Shifting Impairments in Schizophrenia
Source: PLoS One. 2013 Feb 27;8(2):e57257. doi: 10.1371/journal.pone.0057257 (PMC3584128; doi:10.1371/journal.pone.0057257)
Supplement: Table S8 — Correlations between haloperidol-equivalent antipsychotic dose and behavior ([Lose-stay-Lose-shift]) contrasts in Network Components. No significant correlations were observed among antipsychotic doses and shift-evoked neural activity in DMN nodes or ECN nodes. (DOC) [file pone.0057257.s009.doc]

**Table S8. Correlations between haloperidol-equivalent antipsychotic dose and behavior ([Lose-stay – Lose-shift]) contrasts in Network Components**

| **ROI** | |  | **r** |  | |  | **p** |  |
| --- | --- | --- | --- | --- | --- | --- | --- | --- |
| ***Executive Control Network*** | | | | | | | | |
|  | DMPFC | -0.233 | | | 0.224 | | | |
|  | R VLPFC | -0.262 | | | 0.170 | | | |
|  | R DLPFC | 0.044 | | | 0.819 | | | |
|  | L BA6 1 | -0.267 | | | 0.161 | | | |
|  | L BA6 2 | -0.033 | | | 0.867 | | | |
|  |  |  | | |  | | | |
| ***Default Network*** | | | | | | | | |
|  | **L mPFC** | 0.085 | | | 0.662 | | | |
|  | R mPFC | 0.147 | | | 0.447 | | | |
|  | L SFG | 0.032 | | | 0.869 | | | |
|  | R SFG | -0.331 | | | 0.080 | | | |
|  | L TPJ | -0.098 | | | 0.614 | | | |
|  | R TPJ | -0.021 | | | 0.913 | | | |
|  | PCC | -0.357 | | | 0.057 | | | |

Abbreviations: ROI, region of interest; R, right; VS, ventral striatum; L, left; vmPFC, ventromedial prefrontal cortex; ITG, inferior temporal gyrus; PHG, parahippocampal gyrus; PCC, posterior cingulate cortex; DMPFC, dorsomedial prefrontal cortex; DLPFC, dorsolateral prefrontal cortex; BA6, Brodmann Area 6.
