# Supplementary material for: The Roles of Reward, Default, and Executive Control Networks in Set-Shifting Impairments in Schizophrenia
Source: PLoS One. 2013 Feb 27;8(2):e57257. doi: 10.1371/journal.pone.0057257 (PMC3584128; doi:10.1371/journal.pone.0057257)
Supplement: Text S1 — Supporting text and references. (DOC) [file pone.0057257.s010.doc]

**Supporting Text and References for:**

The roles of reward, default, and executive control networks

in set-shifting impairments in schizophrenia

James A. Waltz^1^, Zuzana Kasanova^1^, Thomas J. Ross^2^, Betty J. Salmeron^2^,

Robert P. McMahon^1^, James M. Gold^1^, Elliot A. Stein^2^

1 Maryland Psychiatric Research Center, Department of Psychiatry, University of Maryland School of Medicine, Baltimore, Maryland, USA, 2 Neuroimaging Research Branch, National Institute on Drug Abuse - Intramural Research Program, Baltimore, Maryland, USA

**Supplementary Methods**

*Details on pre-scan instruction and task training.* In a pre-scan training session outside of the MRI scanner, subjects were first presented with a deterministic discrimination to learn and reverse. If a subject did not achieve a discrimination and reversal within 50 trials, then he/she was presented with another exemplar until a discrimination and reversal were both achieved. Once and reversal was achieved, the subject was presented with one or more 90%/10% probabilistic discrimination to learn and reverse, and, finally, one or more 80%/20% probabilistic discriminations. As with the deterministic discrimination: if a subject did not achieve a reversal on any one of the pair-types, then he/she was presented with another exemplar until a reversal was achieved. This training session minimized the number of subjects unable to achieve enough initial discriminations to have sufficient numbers of trials on which they were attempting reversals.

Once in the scanner, subjects were told that they would be presented with a series of pairs of stimuli. In each pair, one stimulus would start out being better than the other, although neither stimulus would always be followed by a reward, or always followed by a punishment, when chosen. Subjects were told that, at some point, the situation would change and the stimulus that was previously the worse stimulus would become the better one, and the stimulus that was previously the better stimulus would become the worse one. Subjects were instructed to try to figure out which of the stimuli was better, and to stick with that stimulus until they decided that the alternate stimulus might be the better one.

*Linear Mixed Effect analyses of event-related MRI data*. For second-level analyses of event-related MRI data, we performed linear mixed effect (LME) analyses of baseline-corrected single-subject average parameter estimates from the first-level analyses, using a modified version of the AFNI script “3dLME”. 3dLME is essentially a front-end to the LME function of the R statistics package (www.r-project.org) [1]. This LME function can be used to perform ANOVA- and GLM-like analyses, but is more flexible in regards to missing data and modeling covariance between measures.

**Supplementary Results**

*Resting-state Functional Connectivity (rsFC) Analyses*. Panels A and B of Figure S1 illustrate clusters in medial PFC, left and right posterior parietal cortex (PPC), left and right superior frontal gyrus, and an extended region of PCC showing significantly correlated activity with the PCC seed in healthy controls. Panels C and D of Figure S1 illustrate clusters in left and right posterior parietal cortex (PPC), left and right superior frontal gyrus, and an extended region of PCC showing significantly correlated activity with the PCC seed in schizophrenia patients. Panels E and F of Figure S1 illustrate clusters in medial PFC, left and right posterior parietal cortex (PPC), left and right superior frontal gyrus, and an extended region of PCC showing significantly correlated activity with the PCC seed in the entire sample.

**References**

1. Pinheiro J, Bates D, DebRoy S, Sarkar D, the R Core team, *NLME: Linear and Nonlinear Mixed Effects Models*, in *R package version 3*. 2008. p. 1-90.
